# The Relationship Between Serum Delta FSH Level and Ovarian Response in IVF/ICSI Cycles

**DOI:** 10.3389/fendo.2020.536100

**Published:** 2020-11-05

**Authors:** Linli Hu, Bo Sun, Yujia Ma, Lu Li, Fang Wang, Hao Shi, Yingpu Sun

**Affiliations:** ^1^Center for Reproductive Medicine, The First Affiliated Hospital of Zhengzhou University, Zhengzhou, China; ^2^Henan Key Laboratory of Reproduction and Genetics, The First Affiliated Hospital of Zhengzhou University, Zhengzhou, China; ^3^Henan Provincial Obstetrical and Gynecological Diseases (Reproductive Medicine) Clinical Research Center, The First Affiliated Hospital of Zhengzhou University, Zhengzhou, China; ^4^Henan Engineering Laboratory of Preimplantation Genetic Diagnosis and Screening, The First Affiliated Hospital of Zhengzhou University, Zhengzhou, China

**Keywords:** IVF, ICSI, controlled ovarian stimulation, recombinant FSH, serum delta FSH level, ovarian response

## Abstract

**Background:**

When ovarian response to FSH stimulation for IVF/ICSI is unsatisfactory, the FSH dose is often adjusted in the treatment cycles, thereby assuming that hormone status and follicular development were insufficient for optimal stimulation.

**Objectives:**

To evaluate whether serum delta FSH levels between D6 of gonadotrophin use and basal serum FSH or between D6 of gonadotrophin use and D1 of gonadotrophin use predict ovarian response in IVF/ICSI cycles.

**Method:**

The participants of this retrospective study were chosen from the Reproductive Medicine, The First Affiliated Hospital of Zhengzhou University between August 2015 and December 2017 (n = 3,109), and during the COS, each participant was given a fixed dose of rFSH in the first 6 days. Delta FSH1: The difference of serum FSH between D6 of gonadotrophin use and basal serum FSH. Delta FSH2: The difference of serum FSH between D6 of gonadotrophin use and D1 of gonadotrophin use. Logistic regression was used to analyze the association between delta FSH1 level and delta FSH2 level and ovarian response. Besides, we also use the tertile statistics to divide the groups.

**Results:**

Part I: Delta FSH1 levels (mean: 1.41 ± 3.46) in normal responders were higher than delta FSH1 levels (mean: 1.07 ± 23.89) in hyper responders (P = 0.0248). The tertile of delta FSH1 is dif ≤ 0, 0 < dif ≤ 2.25 and dif > 2.25. Compared with the hyper responder, the delta FSH1 (0 < dif ≤ 2.25 and dif > 2.25) in the normal responder has a higher ratio and is statistically significant. Part II: Delta FSH2 levels (mean: 4.90 ± 2.84) in normal responders were similar with delta FSH2 levels (mean: 4.74 ± 2.09) in hyper responders (P = 0.103). The tertile of delta FSH1 is dif ≤ 3.91, 3.91 < dif ≤ 5.69 and dif > 5.69. Compared with the hyper responders, the delta FSH2 (3.91 < dif ≤ 5.69 and dif > 5.69) in the normal responders has a higher ratio and is statistically significant.

**Conclusions:**

There is a weak relationship between ovarian response and serum delta FSH levels.

## Introduction

In the controlled ovarian stimulation (COS) for IVF/ICSI, the approach to obtain the optimal ovarian response is still an important topic to be discussed. Although more oocytes were considered to be better over the past few decades, we now aim for an optimal range of 8-15 oocytes (1, 2). Too few oocytes or a poor response is associated with higher rates of treatment cycle cancellation and lower pregnancy rates (3), but too many oocytes or a hyper response is also associated with higher rates of cycle cancellation and an increased risk of ovarian hyperstimulation syndrome (OHSS) (4, 5). To find a direct and non-invasive way to count the primordial follicles before the COS, many studies have been conducted to correctly predict the ovarian response to hyperstimulation (6). Until now, the antral follicle count (AFC) and circulating anti-Müllerian hormone (AMH) were the most accurate methods (7–10). In addition to ovarian reserve status (i.e., antral follicle number), antral follicle sensitivity, and FSH pharmacokinetics influence ovarian response (11), the focus of treatment individualization has been mainly on FSH dose adjustment.

The gonadotropin follicle stimulating hormone (FSH) plays a central role in the regulation of the menstrual cycle and the development of antral follicles (12, 13). For multi-follicular growth, high amounts of exogenous FSH are administered daily to achieve the FSH threshold (14, 15). Currently, recombinant FSH (rFSH) is the most widely used (16, 17), and it has shown bioavailability after single administration of 63–66% (18, 19). Additionally, it will reach the steady state after 5–7 days of repeated administration (20). FSH dose individualization based on an ovarian reserve test could theoretically improve IVF/ICSI treatment outcome. Although we adjusted the FSH doses according to body weight, oestrogen levels, and follicle condition, a range from 100 to 600 IU per day has been used in practice (21), without clear evidence suggesting that such extraordinarily high dosages are effective (22). There is an urgent need for substantiation of this concept by evaluating whether serum FSH levels during stimulation with a fixed FSH dose indeed differ between women with different ovarian responses.

Serum FSH levels measured during controlled ovarian stimulation with rFSH are an adequate reflection of the *in vivo* serum FSH levels to which the ovaries are exposed (23). Because the basal serum FSH level was relatively stable and the rFSH dose was unchanged in the first 6 days of FSH stimulation, the serum delta FSH levels (between D6 of Gn and basal serum FSH or between D6 of Gn and D1 of Gn) could be a more reliable marker for FSH dose adjustment. To our knowledge, no studies have been performed that directly evaluate the relationship between serum delta FSH levels during FSH stimulation and the ovarian response to COS for IVF/ICSI in GnRH agonist cycles. If we assume that serum delta FSH levels can be used to evaluate follicle sensitivity and FSH pharmacokinetics to a certain extent, then we have a better strategy to obtain to ideal number of retrieved oocytes. Moreover, avoiding the use of unnecessary gonadotropin reduces the cost of IVF/ICSI treatment. The aim of this study was therefore to assess whether serum delta FSH levels differ significantly between poor, normal, and hyper responders to a fixed daily dose of rFSH protocol in GnRH agonist cycles.

## Material and Methods

### Study Population

**Figure 1:**
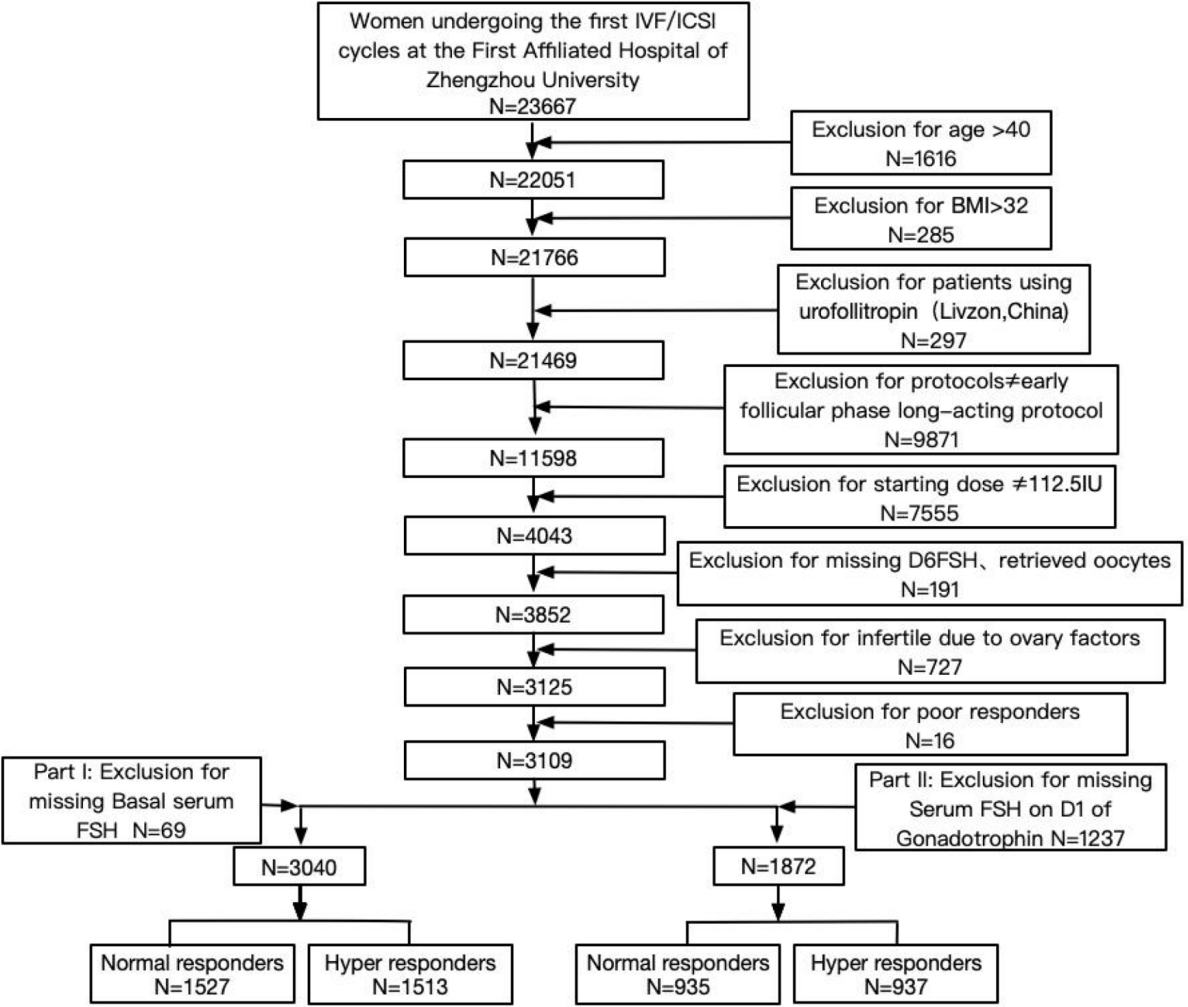
Patient selection flowchart.

From the 23,667 women, 3,109 women were included in this study (**Figure 1**). The present study only includes patients who underwent the first IVF/ICSI-ET cycles in the Reproductive Medicine Center at the First Affiliated Hospital of Zhengzhou University between August 2015 and December 2017. The inclusion criteria were as follows: women aged ≤40 years with a regular menstrual cycle (average cycle length between 21 and 35 days) and an indication for IVF of ICSI. Patients were treated with an early follicular phase long-acting protocol. The rFSH starting dose was 112.5 IU. The exclusion criteria were as follows: body mass index (BMI) > 32 kg/m2, patients with ovarian surgical history, infertility induced by ovarian factors (including PCOS, POI, and endometriosis), and patients using urofollitropin (Livzon, China) in the stimulation cycles. Part I: According to the exclusion of missing basal serum FSH, 3,040 patients were included in the data analysis. Part II: According to the exclusion of missing serum FSH on D1 of gonadotrophin, 1,872 patients were included in the data analysis.

### IVF/ICSI-ET Protocols

After reaching downregulation criteria, all patients were given a 112.5 IU rFSH starting dose. The dose was unchanged for the first 6 days of Gn cycles during ovulation induction. After that, the Gn will be increased or decreased in a timely manner according to the number, size, and growth of follicles. The researchers will determine the increase and decrease in Gn based on the hormone status and follicular development. The criteria for HCG injection include the following: when the diameter of one primary follicle is ≥20 mm and the diameter of the other follicles is ≥18 mm or the quantity of follicles with a diameter of ≥14 mm accounts for more than 2/3 of the follicles. The trigger drug and HCG exposure time will be determined by the researchers according to the patient’s body weight, oestrogen levels, and follicle condition. Eggs will be retrieved 36–37 h after HCG administration.

### Outcome Measure

Part I: Delta FSH1 level (the difference between serum FSH level on D6 of Gn use and basal serum FSH level) was the primary outcome measure.
Part II: Delta FSH2 level (the difference between serum FSH level on D6 of Gn use and serum FSH level on D1 of Gn use) was the primary outcome measure.

### Grouping Method

Step 1: In parts I and II, we used definitions of ovarian response based on GnRH agonist protocols. In accordance with the Bologna criteria, poor response was defined as the retrieval of less than four oocytes (irrespective of oocyte maturity) or cancellation due to poor ovarian response (less than three dominant follicles of >12 mm). Normal response was defined as the retrieval of 4–15 oocytes, and hyper response was defined as the retrieval of more than 15 oocytes or cancellation due to an anticipated risk of OHSS.
Step 2: Because no consensus exists on delta FSH levels, we used tertile statistics in Part I and Part II.

### Statistical Analysis

Data were analysed using the SPSS statistical package (SPSS version 13.0, Chicago, IL). Measurement data are described as the means ± sd, and differences between the groups were compared using Student’s t-test and Wilcox’s test. The categorical variables were compared using a Chi square test or Fisher test, where appropriate. Multivariable statistical analysis was used to assess the relationship between serum delta FSH levels and the number of retrieved oocytes. All data are reported as the mean with their associated standard deviations, and all tests were two-tailed. P < 0.05 was considered statistically significant.

## Results

### Baseline Information and Outcomes of Patients

Of the 23,667 women included, 3,109 women were included in this study (**Figure 1**). Of these women, 16 were categorized as poor responders. Compared to the hyper responders and normal responders, the sample size of poor responders was too small to be discussed. We just analyzed only the hyper and normal groups.

#### Part I

According to the exclusion criteria for missing basal serum FSH, 3,040 patients were included in the data analysis. Of these women, 1,513 were hyper responders, and 1,527 were normal responders. Baseline characteristics for the two response groups are listed in **Table 1**. The ages of the two groups differed significantly. The analysis showed that this result was due to a difference between normal and hyper responders (29.32 ± 3.71 versus 29.00 ± 3.69 years, respectively; P = 0.0087). A significant difference was also found in male age, duration of infertility, BMI, and AFC. Hyper responders had a significantly higher BMI compared to normal responders (22.80 ± 2.56 versus 22.54 ± 2.65, P = 0.0036), and a significantly higher AFC (16.81 ± 5.43 versus 15.12 ± 5.33, P < 0.0001). Hyper responders had a significantly lower total gonadotrophin dose (2058.87 ± 649.98 versus 2141.97 ± 683.31, P = 0.0005) but a significantly higher Gn duration (13.92 ± 1.97 versus 13.56 ± 2.04, P < 0.0001), total oocytes obtained (21.46 ± 5.13 versus 11.17 ± 2.85, P < 0.0001), no. of 2PN oocytes (12.66 ± 5.00 versus 6.87 ± 3.03, P < 0.0001), no. of MII oocytes (16.26 ± 6.38 versus 8.43 ± 3.73, P < 0.0001), and no. of 2PN cleavage embryos (15.11 ± 5.82 versus 8.15 ± 3.26, P < 0.0001).

**Table 1:** Characteristics and outcomes of patients during August 2015 to December 2017.

Variable	PartI		P value	PartII		P value
	Normal responders	Hyper responders		Normal responders	Hyper responders	
N	1527	1513		935	937	
Female age	29.32 ± 3.71	29.00 ± 3.69	0.0087*	29.34 ± 3.65	29.08 ± 3.69	0.0974
Male age	30.59 ± 5.03	30.09 ± 4.73	0.006*	30.57 ± 4.92	30.12 ± 4.48	0.0682
Duration of infertility (years)	3.62 ± 2.52	3.58 ± 2.47	0.9813	3.58 ± 2.47	3.58 ± 2.55	0.9647
Body mass index (kg/m2)	22.54 ± 2.65	22.80 ± 2.56	0.0036*	22.67 ± 2.62	22.97 ± 2.59	0.0124*
Antral follicle count (n.)	15.12 ± 5.33	16.81 ± 5.43	<0.0001*	15.43 ± 5.43	16.97 ± 5.59	<0.0001*
Infertility factors						
Secondary infertility	631 (41.32)	650 (42.96)	0.36851	382 (40.86)	390 (41.62)	0.7362
Primary infertility	895 (58.61)	863 (57.04)		553 (59.14)	547 (58.38)	
Treatment						
ICSI	348 (22.79)	388 (25.64)	0.06619	193 (20.64)	222 (23.69)	0.11208
IVF	1179 (77.21)	1125 (74.36)		742 (79.36)	715 (76.31)	
Total Gonadotrophin dose (IU)	2141.97 ± 683.31	2058.87 ± 649.98	0.0005*	2159.19 ± 677.14	2044.97 ± 645.96	0.0001*
Gonadotrophin duration (d)	13.56 ± 2.04	13.92 ± 1.97	<0.0001*	13.53 ± 2.02	13.87 ± 1.93	<0.0001*
Total oocytes obtained (n.)	11.17 ± 2.85	21.46 ± 5.13	<0.0001*	11.17 ± 2.86	21.45 ± 5.08	<0.0001*
No. of 2PN oocytes	6.87 ± 3.03	12.66 ± 5.00	<0.0001*	6.86 ± 3.05	12.43 ± 4.78	<0.0001*
No. of MII oocytes	8.43 ± 3.73	16.26 ± 6.38	<0.0001*	8.92 ± 3.28	16.89 ± 5.62	<0.0001*
No. of 2PN cleavage embryos	8.15 ± 3.26	15.11 ± 5.82	<0.0001*	8.26 ± 3.26	15.06 ± 5.80	<0.0001*
Outcomes			<0.0001*			<0.0001*
Whole embryo freezing	146 (9.57)	602 (39.79)		87 (9.32)	346 (36.93)	
No cleavage	4 (0.26)	1 (0.07)		3 (0.32)	1 (0.11)	
Cancellation	1 (0.07)	0		0	0	
No insemination	12 (0.78)	3 (0.20)		8 (0.85)	3 (0.32)	
No transfer	12 (0.79)	26 (1.72)		7 (0.75)	10 (1.07)	
No transferred embryo	50 (3.28)	15 (0.99)		30 (3.22)	8 (0.85)	
Transfer	1296 (84.98)	864 (57.11)		796 (85.32)	568 (60.62)	
Abnormal fertilization	4 (0.26)	2 (0.13)		2 (0.21)	1 (0.11)	
Missing	2			2		

*P < 0.05.

#### Part II

According to the exclusion criteria for missing serum FSH on D1 of gonadotrophin, 1,872 patients were included in the data analysis. Of these women, 937 were hyper responders, and 935 were normal responders. Baseline characteristics for the two response groups are listed in **Table 1**. There were no significant differences in female age, male age, and duration of infertility. A significant difference was found in BMI and AFC. Hyper responders in comparison to normal responders had a significantly higher BMI (22.97 ± 2.59 versus 22.67 ± 2.62, P = 0.0124) and a significantly higher AFC (16.97 ± 5.59 versus 15.43 ± 5.43, P < 0.0001). Compared to normal responders, hyper responders had a significantly lower total Gn dose (2044.97 ± 645.96 versus 2159.19 ± 677.14, P = 0.0001) but significantly higher Gn duration (13.87 ± 1.93 versus 13.53 ± 2.02, P < 0.0001), total oocytes obtained (21.45 ± 5.08 versus 11.17 ± 2.86, P < 0.0001), no. of 2PN oocytes (12.43 ± 4.78 versus 6.86 ± 3.05, P < 0.0001), no. of MII oocytes (16.89 ± 5.62 versus 8.92 ± 3.28, P < 0.0001), and no. of 2PN cleavage embryos (15.06 ± 5.80 versus 8.26 ± 3.26, P < 0.0001).

### Serum Hormone Levels

#### Part I

Basal serum FSH levels were significantly lower in normal responders than in hyper responders (P < 0.0001). For normal and hyper responders, the means of serum FSH on D1 of Gn were 3.20 ± 2.50 and 2.96 ± 1.20 mIU/ml (P = 0.0002), respectively. Additionally, the mean of serum FSH on D6 of Gn was significantly higher in normal versus hyper responders (8.03 ± 1.83 versus 7.69 ± 1.87 mIU/ml, P < 0.0001). Hyper responders also had a significantly higher basal serum E2 (44.44 ± 249.38 versus 41.26 ± 43.46 pg/ml, P = 0.0267) and serum E2 on D6 of Gn (165.66 ± 185.35 versus 135.20 ± 156.72 pg/ml, P < 0.0001). No significant differences were found for basal serum LH, basal serum PRL, serum LH on D1 of Gn, serum E2 on D1 of Gn, serum P4 on D1 of Gn, serum LH on D6 of Gn, and serum P4 on D6 of Gn (**Table 2**).

**Table 2:** Hormonal levels of patients during August 2015 to December 2017.

Variable	Part I		P value	Part II		P value
	Normal responders	Hyper responders		Normal responders	Hyper responders	
N	1527	1513		935	937	
Basal serum FSH	6.62 ± 3.26	6.62 ± 23.85	<0.0001*	6.55 ± 3.05	5.94 ± 1.34	<0.0001*
Basal serum LH	5.43 ± 9.51	5.31 ± 2.96	0.0755	5.66 ± 12.11	5.25 ± 2.97	0.5419
Basal serum E2	41.26 ± 43.46	44.44 ± 249.38	0.0267*	41.07 ± 44.67	38.27 ± 27.17	0.2945
Basal serum P4	0.74 ± 1.33	0.73 ± 1.03	0.0433*	0.79 ± 1.57	0.73 ± 1.07	0.3311
Basal serum PRL	20.39 ± 27.31	22.27 ± 66.11	0.6051	20.59 ± 32.28	21.35 ± 45.96	0.1097
Serum FSH on D1 of Gn	3.20 ± 2.50	2.96 ± 1.20	0.0002*	3.20 ± 2.47	2.96 ± 1.19	0.0002*
Serum LH on D1 of Gn	0.63 ± 0.99	0.57 ± 0.36	0.4677	0.62 ± 0.97	0.57 ± 0.36	0.5455
Serum E2 on D1 of Gn	8.31 ± 7.56	7.52 ± 6.30	0.0643	8.26 ± 7.49	7.52 ± 6.28	0.0908
Serum P4 on D1 of Gn	0.48 ± 0.22	0.49 ± 0.21	0.3024	0.48 ± 0.22	0.49 ± 0.21	0.2399
Serum FSH on D6 of Gn	8.03 ± 1.83	7.69 ± 1.87	<0.0001*	8.10 ± 1.90	7.70 ± 1.68	<0.0001*
Serum LH on D6 of Gn	0.51 ± 0.77	0.47 ± 0.95	0.0936	0.49 ± 0.54	0.47 ± 0.54	0.0438*
Serum E2 on D6 of Gn	135.20 ± 156.72	165.66 ± 185.35	<0.0001*	128.44 ± 130.97	168.72 ± 188.31	<0.0001*
Serum P4 on D6 of Gn	0.15 ± 0.20	0.15 ± 0.17	0.4136	0.14 ± 0.19	0.13 ± 0.16	0.7113
Delta FSH1	1.41 ± 3.46	1.07 ± 23.89	0.0248*	/	/	/
Delta FSH2	/	/	/	4.90 ± 2.84	4.74 ± 2.09	0.103

Delta FSH1 = FSH on D6 of gonadotrophin -Basal serum FSH Delta FSH2 = FSH on D6 of gonadotrophin -FSH on D1 of gonadotrophin.

Gn = Gonadotrophin, FSH (mIU/ml), LH (mIU/ml), E2 (pg/ml), P4 (ng/ml), PRL (ng/ml).

*P ＜ 0.05.

#### Part II

Basal serum FSH levels were significantly higher in normal responders than in hyper responders (P < 0.0001). For normal and hyper responders, the means of serum FSH on D1 of Gn were 3.20 ± 2.47 and 2.96 ± 1.19 mIU/ml (P = 0.0002), respectively. Additionally, the mean of serum FSH on D6 of Gn was significantly higher in normal versus hyper responders (8.10 ± 1.90 versus 7.70 ± 1.68 mIU/ml, P < 0.0001). Hyper responders also had a significantly higher serum E2 level on D6 of Gn (128.44 ± 130.97 versus 168.72 ± 188.31 pg/ml, P < 0.0001). No significant differences were found for basal serum LH, basal serum E2, basal serum PRL, serum LH on D1 of Gn, serum E2 on D1 of Gn, serum P4 on D1 of Gn, serum LH on D6 of Gn, and serum P4 on D6 of Gn (**Table 2**).

### Serum Delta FSH

#### Part I

Delta FSH1 levels (mean: 1.41 ± 3.46) in normal responders are higher than delta FSH1 levels (mean: 1.07 ± 23.89) in hyper responders (P = 0.0248) (**Table 2**). The tertile of delta FSH1 is dif ≤ 0, 0 < dif ≤ 2.25 and dif > 2.25. Compared with the normal responder, the delta FSH1(0 < dif ≤ 2.25 and dif > 2.25) has a higher ratio than the hyper responder and is statistically significant (**Table 3**).

**Table 3:** Tertile of Delta FSH1.

Variable	Part I		P value
	Normal responders	Hyper responders	
N	1527	1513	
Delta FSH1			
dif ≤ 0	326 (21.35)	245 (16.19)	0.00094*
0 < dif ≤ 2.25	700 (45.84)	760 (50.23)	
dif > 2.25	501 (32.81)	508 (33.58)	

Delta FSH1 = FSH on D6 of gonadotrophin -Basal serum FSH.

*P ＜ 0.05.

#### Part II

Delta FSH2 levels (mean: 4.90 ± 2.84) in normal responders were higher than delta FSH2 levels (mean: 4.74 ± 2.09) in hyper responders (P = 0.103) (**Table 2**). The tertile of delta FSH1 is dif ≤ 3.91, 3.91 < dif ≤ 5.69 and dif > 5.69. Compared with the hyper responder, the delta FSH2 (3.91 < dif ≤ 5.69 and dif > 5.69) has a higher ratio than the normal responder and is statistically significant (**Table 4**).

**Table 4:** Tertile of Delta FSH2.

Variable	Part II		P value
	Normal responders	Hyper responders	
N	935	937	
Delta FSH2			
dif ≤ 3.91	289 (30.91)	336 (35.86)	0.0268*
3.91 < dif ≤ 5.69	336 (35.94)	288 (30.74)	
dif > 5.69	310 (33.16)	313 (33.4)	

Delta FSH2 = FSH on D6 of gonadotrophin -FSH on D1 of gonadotrophin.

*P < 0.05.

### Correlation

#### Part I

We found a weak but significant correlation between delta FSH1 levels (0 < dif ≤ 2.25) and the number of retrieved oocytes. After adjusting for female age, there was a relationship between ovarian response and the delta FSH1 (0 < dif ≤ 2.25) (OR 1.45; 95% CI, 1.19–1.76). After adjusting for female age, infertility year, basal serum FSH and AFC, there was a relationship between ovarian response and delta FSH1 (0 < dif ≤ 2.25) (OR 1.46; 95% CI, 1.19–1.79) (**Table 5**).

**Table 5:** Association of Delta FSH with ovarian response among patieiits during August 2015 to December 2017.

	case/contiol	OR (95%CD)*	OR (95%a)**
Delta FSHI			
dif ≤ 0	326/245	1.00 (reference)	1.00 (reference)
0 < dif ≤ 2.25	700/760	1.45 (1.19–1.76)	1.46 (1.19–1.79)
dif > 2.25	501/508	1.36 (1.10–1.67)	1.37 (1.10–1.70)
Delta FSH2			
dif ≤ 3.91	289/336	1.00 (reference)	1.00 (reference)
3.91 < dif ≤ 5.69	336/288	0.73 (0.59–0.91)	0.81 (0.63–1.03)
dif > 5.69	310/313	0.86 (0.69–1.08)	1.17 (0.91–1.51)

OR, odds ratio; Cl, confidence interval.

*Adjusted for female age.

**Adjusted for female age, infertility year, basal serum FSH, and Antral follicle count.

#### Part II

We found a weak but significant correlation between delta FSH2 levels (3.91 < dif ≤ 5.69) and the number of retrieved oocytes. After adjusting for female age, there was a relationship between ovarian response and the delta FSH2 (3.91 < dif ≤ 5.69) (OR 0.73; 95% CI, 0.59–0.91). After adjusting for female age, infertility year, basal serum FSH and AFC, there was a relationship between ovarian response and the delta FSH2 (3.91 < dif ≤ 5.69) (OR 0.81; 95% CI, 0.63–1.03) and delta FSH2 (dif > 5.69) (OR 1.17; 95% CI, 0.91–1.51) (**Table 5**).

## Discussion

This study demonstrates that slightly higher delta FSH1 levels and delta FSH2 levels are present in normal responders compared to hyper responders undergoing the first IVF/ICSI cycles at the Reproductive Medicine Center. When performing a multivariable statistical analysis for the number of retrieved oocytes, infertility year, basal serum FSH level and AFC did not appear to play a more important role in determining the response to rFSH stimulation than serum delta FSH levels. A recent study by S.C. Oudshoorn et al. concluded that there is no consistent relationship between ovarian response and serum FSH levels on the day of hCG trigger in a 150 IU fixed dose treatment protocol. Because the studies only focused on serum FSH levels on the day of hCG trigger, it is difficult to directly compare these results to our study (23).

In our study, we divided the women undergoing IVF/ICSI cycles into Part I (missing the basal serum FSH level) and Part II (missing the serum FSH level on D1 of Gn). Normal responders have higher serum FSH levels on D1 of Gn and serum FSH levels on D6 of Gn either in Part I or Part II. One explanation for these results is that normal responders could have antral follicles that can be explained by a different sensitivity or insensitivity of follicles to FSH. FSH stimulates follicular growth by binding to its receptors localized in the granulosa cells of the follicles (24–26). The analysis of patient-specific genotypes might lead to an individualized pharmacogenomic approach to controlled ovarian stimulation (COS). However, no consensus has been established regarding if the genetic variations of these receptors influence serum FSH levels and the degree of ovarian response to stimulation (27–30). Therefore, variation in FSH receptor genotypes is unlikely to be the main reason for the difference in the number of oocytes retrieved in response to standard FSH dose stimulation. Another explanation for this result in normal responders could be that the hyper responders have a higher BMI compared to the normal responders in both Part I and Part II. Several studies have shown that serum FSH levels after administration of rFSH are influenced by the route of administration (intravenous or subcutaneous), body weight and the administered rFSH dose (31–33). Hence, higher BMI in hyper responders could explain why there was no increase in serum FSH level on D1 of Gn and serum FSH level on D6 of Gn in those women.

Currently, there is no research regarding the relationship between serum delta FSH levels and retrieved oocytes. Therefore, there is no consensus for us to reference when grouping the patients by serum delta FSH levels and then to tripartite the women according to the statistical scheme. In Part I, hyper responders have a higher ratio to higher delta FSH1 levels. This finding is inconsistent with the conclusions that normal responders have higher delta FSH1. This result may be due to the inconsistency caused by the individualized differences. However, normal responders have a higher ratio to higher delta FSH2 levels, which is consistent with the conclusions that normal responders have higher delta FSH2. Serum delta FSH levels differed conversely between the response groups than we had hypothesized when designing the study. This finding may seem to be in contrast to these studies, suggesting that a higher dose of FSH leads to higher serum FSH levels and a slightly greater oocyte yield (22, 34). We must draw a conclusion carefully because this study lacks data from poor responders.

Most previous studies illustrate that 8–15 oocytes give a pregnancy rate plateau, and there seems to be no benefit of creating an excessive response with only higher risks of jeopardizing the patients’ health and increasing costs of IVF/ICSI treatment (35). Even though doctors have paid attention to the problem, approximately 20% of women undergoing IVF/ICSI experience an excessive response (36), and up to 7% may develop OHSS. In the stimulation protocol, researchers will determine the increase and decrease in Gn based on the hormone status and follicular development after D6 of Gn. Even if the hyper responders use a lower total gonadotrophin dose, 40% of patients still underwent whole embryo freezing because of the high ovarian response. The serum delta FSH level can be an additional marker to guide the adjustment of the rFSH dose in the agonist cycles.

The present study has several strengths. First, to increase the reliability of the results, all blood samples from patients were collected at approximately the same point in the experiments. Moreover, the rFSH used was the same, and the rFSH dose was unchanged in the first six days of FSH stimulation. Additionally, several limitations should be mentioned. While we excluded women with a different FSH starting dose, a dose adjustment during the treatment cycle could possibly affect the number of oocytes obtained. Compared to the normal and hyper responders, the sample size of the poor responders was too small to be discussed.

In general, the results of this study show that there is a weak relationship between ovarian response and serum delta FSH levels in the rFSH fixed dose treatment protocol. This finding may imply that decreasing the dose of rFSH in women who respond highly will lead to a more ideal oocyte yield and improve the safety of IVF/ICSI treatment. However, these issues should be studied in a larger trial with poor responders and a true dose comparison design.

## Data Availability Statement

The datasets generated for this study are available on request to the corresponding author.

## Ethics Statement

The studies involving human participants were reviewed and approved by Research Ethics Committee of the First Affiliated Hospital of Zhengzhou University. The patients/participants provided their written informed consent to participate in this study.

## Author Contributions

LH performed the original trial. LH and BS designed the study. LH carried out all necessary data analyses and in collaboration with BS wrote the article. YM, FW, and HS collected all data. LL revised the article. All authors participated in the interpretation of the data and provided significant revisions. YS read and approved the final version of the article. All authors contributed to the article and approved the submitted version.

## Funding

This work was funded by the Chinese Medical Association Clinical Medical Research Special Fund Project (FDN-17020190688), the Medical Science and Technology Research Project Joint Co-construction Project of Henan Province (FDN-2018020116), and the Henan Provincial Higher Education Key Research Project Plan (FDN-19A320056).

## Conflict of Interest

The authors declare that the research was conducted in the absence of any commercial or financial relationships that could be construed as a potential conflict of interest.
